# Albumin-to-alkaline phosphatase ratio serves as a prognostic indicator in unresectable pancreatic ductal adenocarcinoma: a propensity score matching analysis

**DOI:** 10.1186/s12885-020-07023-9

**Published:** 2020-06-09

**Authors:** Ke Zhang, Shu Dong, Yan-Hua Jing, Hui-Feng Gao, Lian-Yu Chen, Yong-Qiang Hua, Hao Chen, Zhen Chen

**Affiliations:** 1 Department of Integrative Oncology, Fudan University Shanghai Cancer Center, Shanghai, 200032 China; 2 Department of Oncology, Shanghai Medical College, Fudan University, Shanghai, 200032 China

**Keywords:** Pancreatic ductal adenocarcinoma, Albumin-to-alkaline phosphatase ratio, Propensity score matching, Overall survival, Prognostic marker

## Abstract

**Background:**

Recent evidence suggests that albumin-to-Alkaline Phosphatase Ratio (AAPR) functions as a novel prognostic marker in several malignancies. However, whether it can predict the prognosis of unresectable pancreatic ductal adenocarcinoma (PDAC) remains unclear. Herein, we seek to investigate this possibility by a propensity score matching (PSM) analysis.

**Methods:**

This was a retrospective cohort study in which 419 patients diagnosed with unresectable PDAC and receiving chemotherapy were recruited. Patients were stratified based on the cutoff value of AAPR. The PSM analysis was performed to identify 156 well-balanced patients in each group for overall survival (OS) comparison and subgroup analysis. Univariate and multivariate analyses were carried out to examine the potential of AAPR to indicate the prognosis of unresectable PDAC. The prediction performance of conventional model and combined model including AAPR was compared using the Akaike Information Criterion (AIC) and concordance index (C-index).

**Results:**

We identified an AAPR of 0.4 to be the optimal cutoff for OS prediction. Patients with AAPR≤0.4 had significantly shorter OS compared with patients with AAPR> 0.4 (6.4 versus 9.3 months; *P* < 0.001). Based on the PSM cohort and entire cohort, multivariate Cox analysis revealed that high pretreatment for AAPR was an independent marker predicting favorable survival in unresectable PDAC (hazard ratio, 0.556; 95% confidence interval, 0.408 to 0.757; *P* < 0.001). Significant differences in OS were observed in all subgroups except for the group of patients age ≤ 60. Combined prognostic model including AAPR had lower AIC and higher C-index than conventional prognostic model.

**Conclusions:**

Pretreatment AAPR servers as an independent prognostic indicator for patients with unresectable PDAC. Inclusion of AAPR improved the prediction performance of conventional prognostic model, potentially helping clinicians to identify patients at high risk and guide individualized treatment.

## Background

Pancreatic ductal adenocarcinoma (PDAC) is a fatal disease and the fourth leading cause of cancer-related death worldwide with a 5-year survival rate of 8% [1]. In China, PDAC is ranked sixth among cancer-related deaths and ninth among the most prevalent cancers [2]. Currently, there are no effective treatments for PDAC. Surgical resection remains the only curative treatment. However, the overall management of PDAC patients is far from being satisfactory, since approximately 80% of patients are diagnosed at advanced and unresectable stages and received systemic chemotherapy [3, 4]. Gemcitabine-based combination regimen or the fluorouracil, leucovorin, irinotecan, and oxaliplatin (FOLFIRINOX) are currently the most recommended in cases of advanced PDAC [5–7]. Unfortunately, tumor biology of PDAC causes resistance to systemic therapy, thereby leading to poor prognosis. Notably, patients with similar PDAC disease conditions sometimes have different outcomes. Therefore, it is crucial to identify a prognostic marker that can predict survival and hence guide clinical decisions for patients with unresectable PDAC.

Serum albumin (ALB) is synthesized in the liver and is the most abundant protein in plasma and this may explain why it is usually included in standard blood tests to assess hepatic function. Also, it performs various roles, ranging from regulating oncotic pressure to scavenging reactive oxygen species. Recent evidence has shown that ALB is an accurate biomarker reflecting underlying host systemic inflammatory responses [8]. Beside that, alkaline phosphatase (ALP) is enriched in kidney, bile duct, and liver [9]. Studies have shown that serum ALP is a useful marker of tumor growth and metastasis [10–12].

Accordingly, albumin-to-alkaline phosphatase ratio (AAPR) is postulated to be a prognostic indicator in hepatocellular carcinoma [13]. So far, its prognostic performance has been studied for multiple malignancies such as non-small-cell lung cancer [14], metastatic nasopharyngeal cancer [15], cholangiocarcinoma [16], upper tract urothelial carcinoma [17], renal cell carcinoma [18], and cervical cancer [19]. Notably, Pu et al. [20] concluded that preoperative AAPR is an independent factor that determines PDAC progression after curative resection. However, little is known about the ability of AAPR to reflect the clinical outcomes of unresectable PDAC, which accounts for the majority of the PDAC.

Taking this need into consideration, we conducted a retrospective cohort study and performed propensity score-matching (PSM) analysis to investigate the prognostic significance of pretreatment AAPR in unresectable PDAC treated with chemotherapy.

## Methods

### Study design and patient characteristics

Prior to this study, approval was obtained from the Ethics Committee of Fudan University Shanghai Cancer Center. All participants were enrolled retrospectively and provided written informed consent in line with our institutional guidelines.

This was a single-center retrospective cohort study. A total of 419 patients pathologically diagnosed with unresectable PDAC and treated at Fudan University Shanghai Cancer Center between January 1st, 2011 and December 31st, 2015 were enrolled. During participant enrollment, the following criteria were employed: 1) patients diagnosed with pancreatic adenocarcinoma histologically or cytologically; 2) subjects who had metastasized and locally advanced disease or defined as stage III or IV in line with the American Joint Committee on Cancer, 8th edition (Chicago, IL, USA) [21]; 3) patients who had no history of malignant disease prior to treatment. Participants meeting the following criteria were precluded from the study: 1) patients who had untreated obstructive jaundice, acute infectious diseases, or hematological diseases; 2) patients who lacked complete clinicopathological and follow-up data.

Data on conventional clinicopathological variables, such as demographic data, tumor location, stage, carbohydrate antigen 19–9 (CA19–9), ALB, and ALP, were retrieved. We performed all biochemical tests prior to treatment and diagnosis. This information was retrieved from electronic record system at our center.

The primary outcome of this study was overall survival (OS), which was defined as the duration from diagnosis to date of last follow-up or death due to any cause. Patients follow-up information was obtained via medical records or telephone interviews. The last follow-up date was December 31st, 2017.

### Albumin-to-alkaline phosphatase ratio (AAPR)

Pretreatment AAPR was defined as a serum albumin/serum alkaline phosphatase ratio. The median AAPR for the whole cohort was 0.46 (range, 0.05 to 1.60). We further used the X-tile 3.6.1 software (Yale University, New Haven, CT) [22] to determine the optimal cutoff point of AAPR for OS prediction in the entire cohort. Consequently, all patients were stratified into the low AAPR (≤0.4) group and the high AAPR (> 0.4) group for subsequent analyses (Figure S1).

We then compared the baseline patient characteristics of low and high AAPR groups. Moreover, subgroup analyses were conducted according to patients’ age, sex, location, tumor stage, and CA19–9 level.

### Statistical analysis

Pearson’s chi-square test and Fisher’s exact test were utilized to compare baseline characteristics between groups. The survival estimates of OS were determined using the Kaplan-Meier method, and group differences were analyzed using log-rank test. Univariate and multivariate analyses were carried out using the Cox proportional hazards regression model to evaluate the prognostic effect of variables and estimate the hazard ratio (HR) with a 95% confidence interval (CI).

Propensity score matching (PSM) analysis was applied to reduce bias by equating the two groups based on the following variables: age, sex, location, tumor stage, and CA19–9 level. In the PSM analysis, the confounders must not be influenced by factors of AAPR, indicating that ALB and ALP were not included for balance [23]. The PSM analysis was performed using the nearest-neighbor 1:1 matching method with a caliper width of 0.02 of the pooled standard deviation of the logit of the propensity score. Covariate balance plot and histogram of propensity score were generated to assess balance after matching.

The prediction performance of conventional model and combined model including AAPR was compared using the Akaike Information Criterion (AIC) [24] and the concordance index (C-index) [25]. Given a set of candidate models, the preferred model is the one that has the minimum AIC. Higher C-index represents better discrimination power of the model.

All tests were two-sided and were carried out with IBM SPSS 24.0 software (IBM SPSS Statistics, Version 24.0. Armonk, NY) and R software [26], version 3.5.0 (https://www.r-project.org/). The R package MatchIt [27] was applied to perform PSM analysis. Covariates balance plot was generated by R package cobalt [28]. The stats package, part of R software, was called to obtain AIC. The R packages Hmisc [29] was used to calculate the C-index of prediction models. *P*-value < 0.05 was considered statistically significant.

## Results

### Patient characteristics

**Fig. 1:**
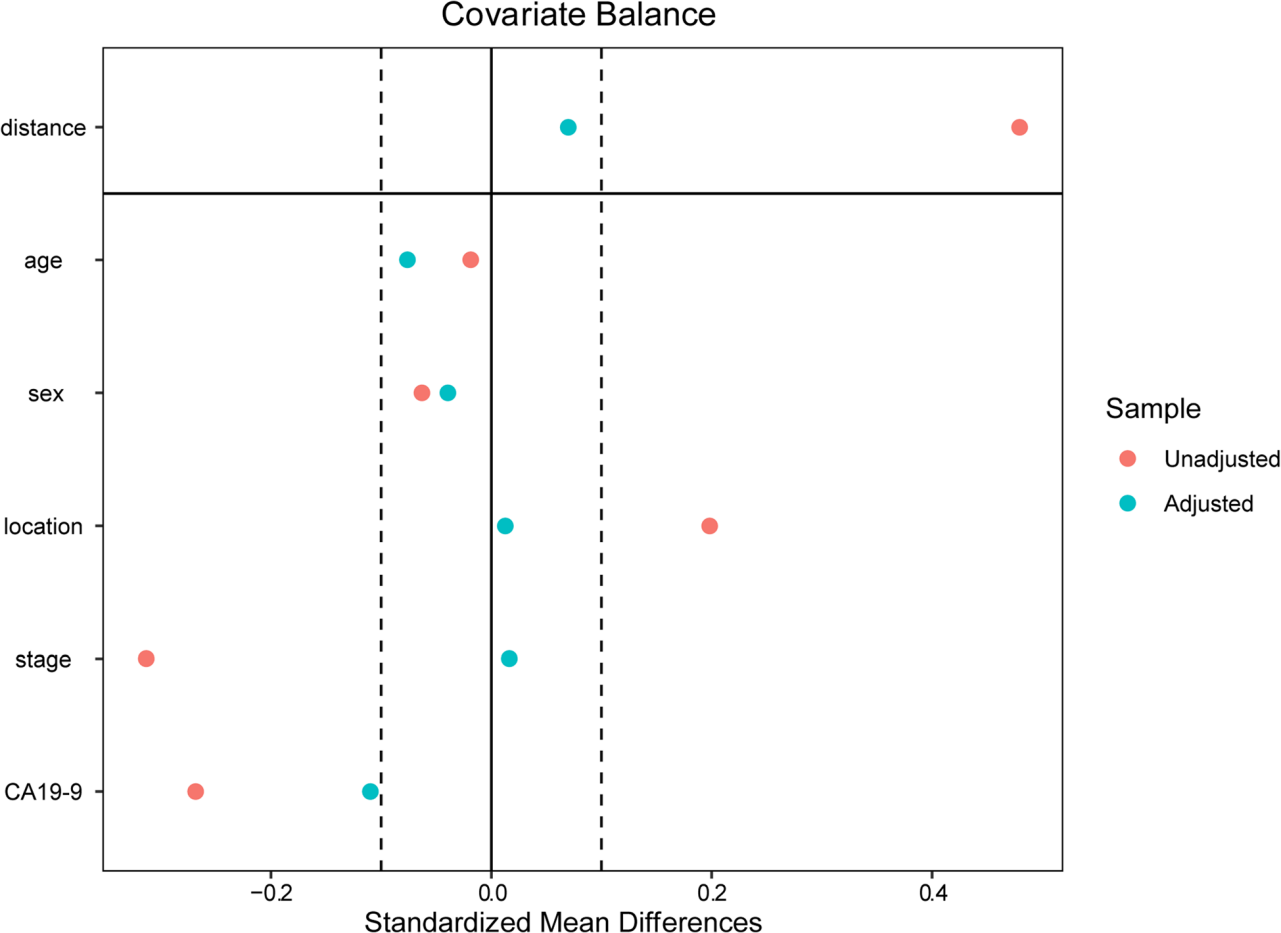
Covariate balance plot for assessing balance between low and high AAPR groups after PSM

This respective study covered 419 patients with unresectable PDAC. These patients were followed up for a median period of 8 months (range, 1 to 42 months). Six patients were alive at the last follow-up. All subjects received gemcitabine-based palliative chemotherapy (gemcitabine monotherapy or combination regimens, as exemplified by gemcitabine plus albumin-bound paclitaxel, gemcitabine plus capecitabine, and gemcitabine plus oxaliplatin/ cisplatin). The baseline clinicopathologic features of the patients according to the APPR level before and after PSM are presented in Table 1. In the unmatched cohort, patients with low APPR levels were associated with a more advanced tumor stage and higher CA19–9 level. After PSM, there were 156 matched pairs in two groups. Among these patients, 195 (62.5%) were male and 117 (37.5%) female, with a median age of 62 years (range, 25 to 83 years). Also, 50 (16.0%) patients presented with stage III of the disease, whereas the remaining 262 (84.0%) presented with stage IV. Covariate balance plot and histogram of propensity score showed that baseline characteristics in the low and high AAPR groups were well matched after PSM (Fig. 1, Figure S2).

**Table 1:** Baseline patient information and clinical features

Characteristics	Before PSM				After PSM			
	Total (*N* = 419)	AAPR≤0.4 (*N* = 173)	AAPR> 0.4 (*N* = 246)	P-value	Total (*N* = 312)	AAPR≤0.4 (*N* = 156)	AAPR> 0.4 (*N* = 156)	*P*-value
Age								
≤ 60	200 (47.7)	78 (45.1)	122 (49.6)	0.373	136 (43.6)	72 (46.2)	64 (41.0)	0.424
> 60	219 (52.3)	95 (54.9)	124 (50.4)		176 (56.4)	84 (53.8)	92 (59.0)	
Sex								
Male	269 (64.2)	108 (62.4)	161 (65.4)	0.536	195 (62.5)	99 (63.5)	96 (61.5)	0.815
Female	150 (35.8)	65 (37.6)	85 (34.6)		117 (37.5)	57 (36.5)	60 (38.5)	
Tumor location								
Head	177 (42.2)	83 (48.0)	94 (38.2)	0.056	137 (43.9)	66 (42.3)	71 (45.5)	0.648
Body/tail	242 (57.8)	90 (52.0)	152 (61.8)		175 (56.1)	90 (57.7)	85 (54.5)	
Stage								
III	83 (19.8)	22 (12.7)	61 (24.8)	0.003	50 (16.0)	22 (14.1)	28 (17.9)	0.441
IV	336 (80.2)	151 (87.3)	185 (75.2)		262 (84.0)	134 (85.9)	128 (82.1)	
CA19–9 (U/mL)								
≤ 1000	247 (58.9)	85 (49.1)	162 (65.9)	0.001	170 (54.5)	85 (54.5)	85 (54.5)	1.000
> 1000	172 (41.1)	88 (50.9)	84 (34.1)		142 (45.5)	71 (45.5)	71 (45.5)	
ALB (g/L)								
≤ 35	26 (6.2)	20 (11.6)	6 (2.4)	< 0.001	20 (6.4)	17 (10.9)	3 (1.9)	0.002
> 35	393 (93.8)	153 (88.4)	240 (97.6)		292 (93.6)	139 (89.1)	153 (98.1)	
ALP (U/L)								
≤ 125	296 (70.6)	51 (29.5)	245 (99.6)	< 0.001	204 (65.4)	49 (31.4)	155 (99.4)	< 0.001
> 125	123 (29.4)	122 (70.5)	1 (0.4)		108 (34.6)	107 (68.6)	1 (0.6)	

*Abbreviations*: *ALP* alkaline phosphatase, *ALB* albumin, *AAPR* albumin to alkaline phosphatase ratio, *HR* hazard ratio, *CI* confidence interval, *CA19–9* carbohydrate antigen 19–9, *PSM* propensity score matching, *NA* not available

### Association between AAPR and clinicopathologic characteristics in unresectable PDAC

The clinicopathologic features of high and low AAPR groups are summarized in Table 1. Subjects with low AAPR values had significantly higher ratios of stage IV disease (*P* = 0.003), high CA19–9 level (*P* = 0.001), hypoalbuminemia (*P* < 0.001), and elevated ALP (*P* < 0.001) compared with those patients with high AAPR levels.

### Prognostic significance of AAPR in unresectable PDAC

**Fig. 2:**
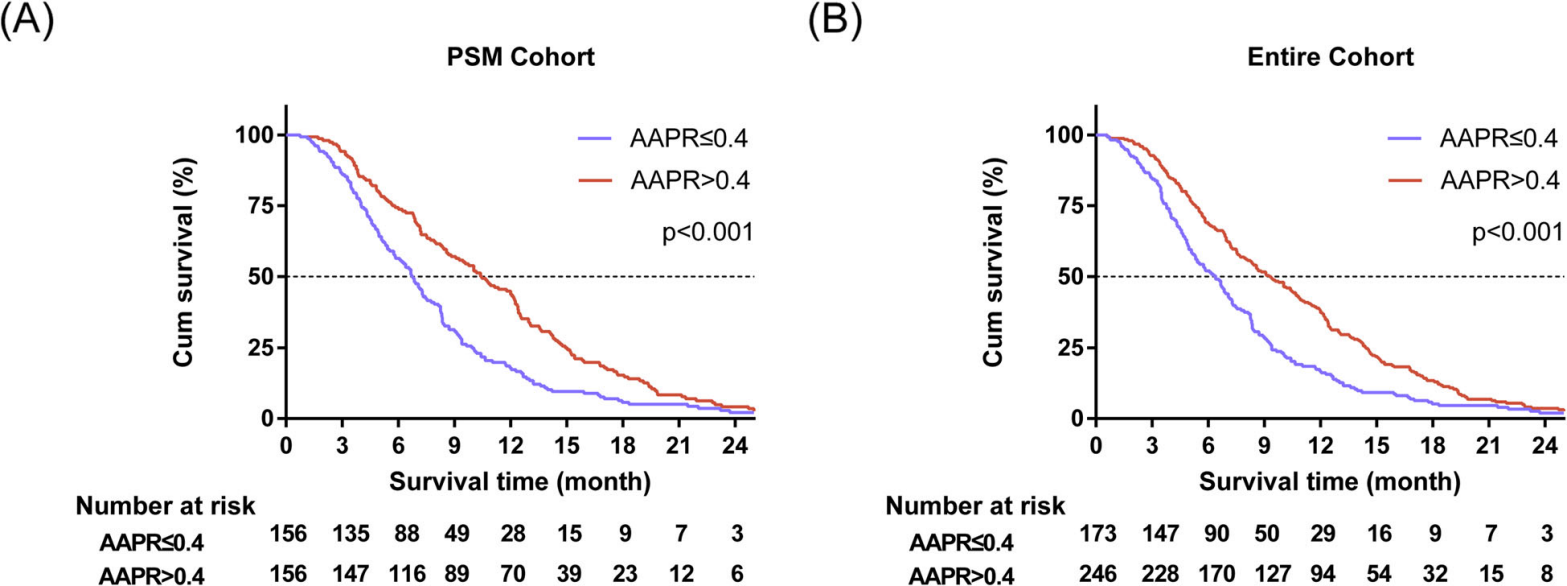
Kaplan-Meier survival curves for OS of subjects with unresectable PDAC based on AAPR levels in the entire cohort (**a**) and PSM cohort (**b**)

In the entire cohort, the Kaplan-Meier analysis suggested that low AAPR positively correlated with poor survival (*p* < 0.001, Fig. 2a). The median OS of subjects with AAPR≤0.4 and AAPR> 0.4 was 6.4 months (95% CI, 5.4 to 7.4) and 9.3 months (95% CI: 7.9–10.6), respectively. Univariate Cox analysis revealed that the tumor stage, CA19–9 level, ALP, and AAPR were strongly linked to OS (Table 2). Furthermore, it was uncovered that AAPR was an independent prognostic factor of OS (HR, 0.556; 95% CI, 0.408 to 0.757; *P* < 0.001; Table 3).

**Table 2:** Univariate Cox regression analysis of clinical parameters for OS

Characteristics	Before PSM		After PSM	
	HR (95% CI)	*P*-value	HR (95% CI)	*P*-value
Age, years (≤60 vs. > 60)	1.064 (0.877–1.292)	0.528	0.995 (0.794–1.248)	0.968
Sex (male vs. female)	1.124 (0.919–1.376)	0.254	1.157 (0.918–1.458)	0.218
Tumor location (head vs. body/tail)	0.872 (0.717–1.062)	0.872	1.036 (0.825–1.302)	0.760
Stage (III vs. IV)	1.643 (1.278–2.113)	< 0.001	1.438 (1.046–1.976)	0.025
CA19–9, U/mL (≤1000 vs. > 1000)	1.472 (1.204–1.800)	< 0.001	1.416 (1.123–1.785)	0.003
ALB, g/L (≤35 vs. > 35)	0.670 (0.446–1.005)	0.053	0.713 (0.448–1.136)	0.155
ALP, U/L (≤125 vs. > 125)	1.299 (1.049–1.608)	0.016	1.210 (0.955–1.533)	0.114
AAPR (≤0.4 vs. > 0.4)	0.624 (0.512–0.761)	< 0.001	0.599 (0.477–0.753)	< 0.001

*Abbreviations*: *AAPR* albumin to alkaline phosphatase ratio, *ALB* albumin, *ALP* alkaline phosphatase, *CA19–9* carbohydrate antigen 19–9, *CI* confidence interval, *HR* hazard ratio, *PSM* propensity score matching

**Table 3:** Multivariate Cox regression analysis of clinical parameters for OS

Characteristics	Before PSM		After PSM	
	HR (95% CI)	*P*-value	HR (95% CI)	*P-*value
Stage (III vs. IV)	1.372 (1.053–1.787)	0.019	1.200 (0.865–1.666)	0.274
CA19–9, U/mL (≤1000 vs. > 1000)	1.296 (1.053–1.594)	0.014	1.356 (1.071–1.717)	0.011
ALP, U/L (≤125 vs. > 125)	0.743 (0.537–1.028)	0.073	NA	NA
AAPR (≤0.4 vs. > 0.4)	0.556 (0.408–0.757)	< 0.001	0.619 (0.491–0.779)	< 0.001

*Abbreviations*: *NA* not available, *ALP* alkaline phosphatase, *CA19–9* carbohydrate antigen 19–9, *HR* hazard ratio, *CI* confidence interval, *PSM* propensity score matching, *AAPR* albumin to alkaline phosphatase ratio

In the PSM cohort, the KM analysis showed that patients with AAPR≤0.4 had shorter OS compared with patients with AAPR> 0.4 (P < 0.001, Fig. 2b). The median OS of high and low AAPR groups was 10.4 months (95% CI, 8.6 to 12.2), and 6.7 months (95% CI, 5.9 to 7.5), respectively. Results of the univariate Cox analysis suggested that the tumor stage, CA19–9 level, and AAPR had prognostic value for survival. High AAPR was significantly related to longer OS (HR, 0.599; 95% CI, 0.477 to 0.753; *P* < 0.001; Table 2). Finally, the multivariate Cox analysis demonstrated that AAPR might be an independent prognostic indicator for OS (HR, 0.619; 95% CI, 0.491 to 0.779; *P* < 0.001; Table 3).

### Subgroup analysis

**Fig. 3:**
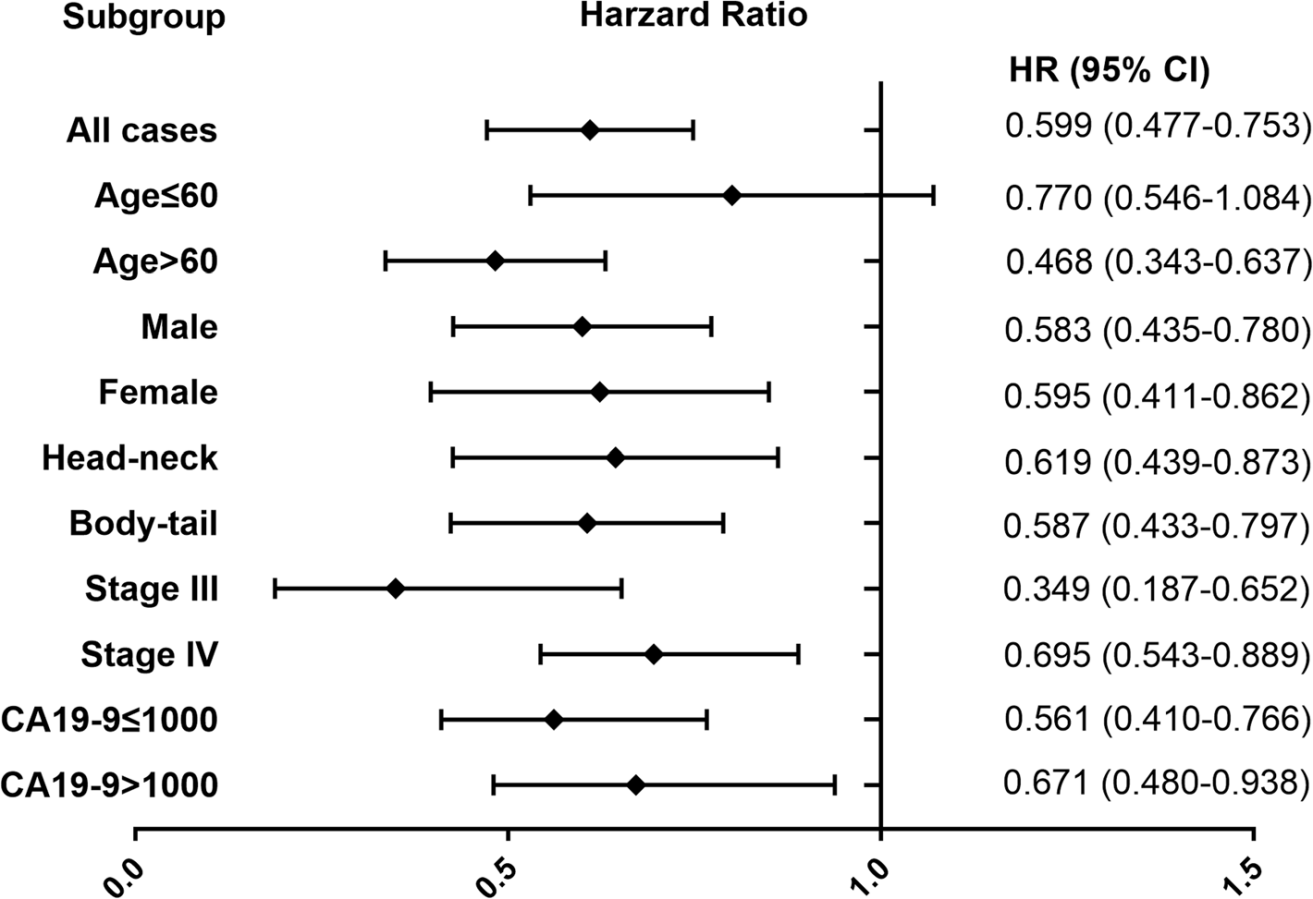
Subgroup analyses of the predictive value of AAPR for overall survival. Hazard ratios (HR) of AAPR levels were calculated for overall survival in different patient subgroups in the PSM cohort

We performed subgroup survival analyses to further assess the prognostic performance of AAPR in unresectable PDAC patients. This was done by grouping patients according to baseline characteristics, such as sex, age, tumor location, tumor stage, and CA19–9 level. We found that pretreatment AAPR significantly associated with OS in all subgroups except for the group of age ≤ 60 (Fig. 3).

### Prognostic models evaluation

Based on the multivariate Cox analysis, the conventional prognostic model included tumor stage, CA19–9 level. The combined model was constructed based on the conventional model and AAPR. These two prognostic models were evaluated by AIC and C-index (Table 4). The model with lower AIC was the combined model including AAPR in both total cohort and PSM cohort. In addition, the model with higher C-index for both total cohort and PSM cohort was also the combined model with AAPR included.

**Table 4:** Prognostic model comparison for the conventional model and combined model including AAPR

	Total cohort	PSM cohort
Conventional model (AIC)	4153.24	2921.76
Combined model (AIC)	4150.64	2914.30
Conventional model [C-index (95% CI)]	0.616 (0.585–0.647)	0.595 (0.553–0.632)
Combined model [C-index (95% CI)]	0.626 (0.595–0.657)	0.619 (0.584–0.654)

*Abbreviations*: *AIC* Akaike Information, *CI* confidence interval, *C-index* concordance index, *PSM* propensity score matching

## Discussion

In the current study, we constructed AAPR, an easily available index comprised of serum ALB and ALP level. We further investigated its prognostic significance in unresectable PDAC patients. Analysis of the patient characteristics showed that low AAPR was closely associated with more advanced stage and higher CA19–9 level. PSM analysis was performed to generate well-balanced patients in low and high AAPR groups for survival comparison. Univariate and multivariate Cox analysis indicated that APPR served as an independent prognostic predictor for OS in both entire cohort and PSM cohort. In addition, we found that AAPR could be used in most subgroups except for the group of age ≤ 60. More importantly, inclusion of AAPR improved the prediction performance of conventional prognostic model.

To our best knowledge, Chan et al. [13] were the first to report that AAPR can act as a novel prognostic index for patients with hepatocellular carcinoma patients underwent curative surgery. Since then, numerous studies have looked into the prognostic performance of AAPR for various malignancies, such as metastatic nasopharyngeal cancer [15], non-small-cell lung cancer [14], cholangiocarcinoma [16], upper tract urothelial carcinoma [17], renal cell carcinoma [18], and cervical cancer [19]. Particularly, Pu et al. [20] examined the AAPR in patients receiving surgery for PDAC and concluded that preoperative AAPR could independently predict postoperative OS in PDAC patients. Consistently, our data revealed that low AAPR correlated with poor OS. Also, we revealed that AAPR might function as an independent prognostic predictor in unresectable PDAC patients. In the subgroup analyses, the outcome of survival consistently favored the high AAPR group across most subgroups except in the group of age ≤ 60. Despite the findings not being significant, the trend in the group of age ≤ 60 was consistent with other groups. In addition, it was reported that patients with low AAPR had less favorable pathological features, including higher rations of lymph node metastasis and advanced T stage in non-small-cell lung cancer [14]. Low AAPR was reported to be associated with larger tumor size and more lymph node positive status in upper tract urothelial carcinoma [17]. Similarly, we found that patients with low APPR levels were more likely to have metastatic disease and higher CA19–9 level. These findings suggested that low AAPR was associated with progress of disease, which called for attention from clinicians.

Cancer is marked by rapid tumor cell proliferation and invasion [30], which may lead to various metabolic changes, such as enhanced production of some serum proteins, cytokines, and hormones. Thus, serum ALB and ALP might reflect tumor progression, and can, therefore, be used to predict clinical outcomes. The following mechanisms may explain the biological relationship between AAPR and prognosis of unresectable PDAC.

As the most abundant protein in serum, ALB is the primary determinant of plasma oncotic pressure. Besides, ALB offers storages and conveyors for many exogenous and endogenous substances [31]. Serum ALB levels reflect the nutritional status and liver function in humans. It is widely recognized that inflammation, tumor microenvironment, and their interaction play critical roles in tumor progression [32]. Studies have shown that ALB can modulate the inflammatory reaction by binding prostaglandins, lipopolysaccharide, and reactive oxygen species [33]. Moreover, ALB has been found to suppress the cell cycle and progression of hepatocellular carcinoma [34, 35]. Recent lines of evidence have identified ALB as an independent prognostic indicator in various malignant tumors, including lung [36], breast [37], and gastric cancers [38].

As a phosphate monoester hydrolase, ALP promotes the hydrolysis and transfer of phosphate groups in alkaline conditions [39]; therefore, it is regularly examined to evaluate liver function in clinical practice. Increasing evidence has shown that ALP can serve as a tumor-associated antigen and biomarker of cancer cell proliferation [11, 40]. On the contrary, a reduction of ALP activity induces cell death, mesenchymal-to-epithelial transition, and suppresses cell migration [41]. Besides, ALP activity level is commonly used to predict bone metastasis in various types of cancer [42, 43]. In the present study, patients with lower AAPR displayed poor prognosis, an observation that was consistent with the previous findings.

As a result of inflammation, oxidative stress produces reactive oxygen species that can damage protein, lipids, DNA, and facilitate the production of highly mutagenic metabolites [44]. Accordingly, oxidative stress plays a vital role in the tumorigenesis of PDAC [45]. Elevated ALP levels may serve as a potential predictor of oxidative stress [46], thereby contributing to cancer progression and poor disease outcomes [47, 48]. Because of this, patients with low AAPR might have a poor prognosis.

Herein, we demonstrated the prognostic value of AAPR for unresectable PDAC by multivariate and univariate models as well as PSM analyses. Besides, patients’ characteristics were well matched to minimize potential confounding bias. The prognostic value of AAPR was investigated in the entire cohort and further validated in the PSM cohort. Moreover, AAPR was confirmed to be associated with OS in most subgroups, which suggested that AAPR could be applied in patients with different clinicopathological features.

However, this study had a few limitations. First, our cohort was a retrospective cohort in a single center and composed of Chinese patients only; therefore, these results might not apply to other populations. Further multicenter prospective studies are thus recommended. Second, FOLFIRINOX is a standard regimen option for patients with metastasized PDAC. However, FOLFIRINOX is not widely used by Chinese patients because of its severe side effects. Whether AAPR could be used in patients receiving FOLFIRINOX remains unclear. Third, Karnofsky performance scale (KPS) [49] and tumor size [50] were reported as prognostic factors in advanced pancreatic cancer receiving chemotherapy and were not analyzed in this study. The potential confounding effect of these factors on AAPR needs further assessment. Forth, the underlying mechanism between the AAPR and cancer biology was not investigated and therefore needs further research.

## Conclusions

In conclusion, our results suggest that pretreatment AAPR may serve as a prognostic predictor of overall survival in patients with unresectable PDAC. AAPR shows potential in improving the prediction performance of conventional prognostic model, which may help clinicians to identify patients at high risk and guide individualized treatment. Further prospective studies are needed to validate these results.

## Supplementary information

**Additional file 1: Figure S1**. X-tile software for determining the optimal cutoff of AAPR. Chi-square test (A), Histogram of AAPR (B), Kaplan-Meier analysis (C), Relative risk plot (D).

**Additional file 2: Figure S2**. Mirrored histogram showing distribution and overlapping of propensity score between low and high AAPR groups in unmatched (A) and matched (B) cases.

## Data Availability

All analysed data during the current study were presented in the main manuscript and supplementary file. The original datasets are available from the corresponding author on reasonable request.

## Abbreviations

AAPR: Albumin-to-Alkaline Phosphatase Ratio
AIC: Akaike Information Criterion
ALB: Albumin
ALP: Alkaline phosphatase
CA19–9: Carbohydrate antigen 19–9
CI: Confidence interval
C-index: Concordance index
FOLFIRINOX: Fluorouracil, leucovorin, irinotecan, and oxaliplatin
HR: Hazard ratio
KPS: Karnofsky performance scale
OS: Overall survival
PDAC: Pancreatic ductal adenocarcinoma
PSM: Propensity score matching
