# Supplementary figures and images for: Albumin-to-alkaline phosphatase ratio serves as a prognostic indicator in unresectable pancreatic ductal adenocarcinoma: a propensity score matching analysis
Source: BMC Cancer. 2020 Jun 9;20:541. doi: 10.1186/s12885-020-07023-9 (PMC7285790; doi:10.1186/s12885-020-07023-9)

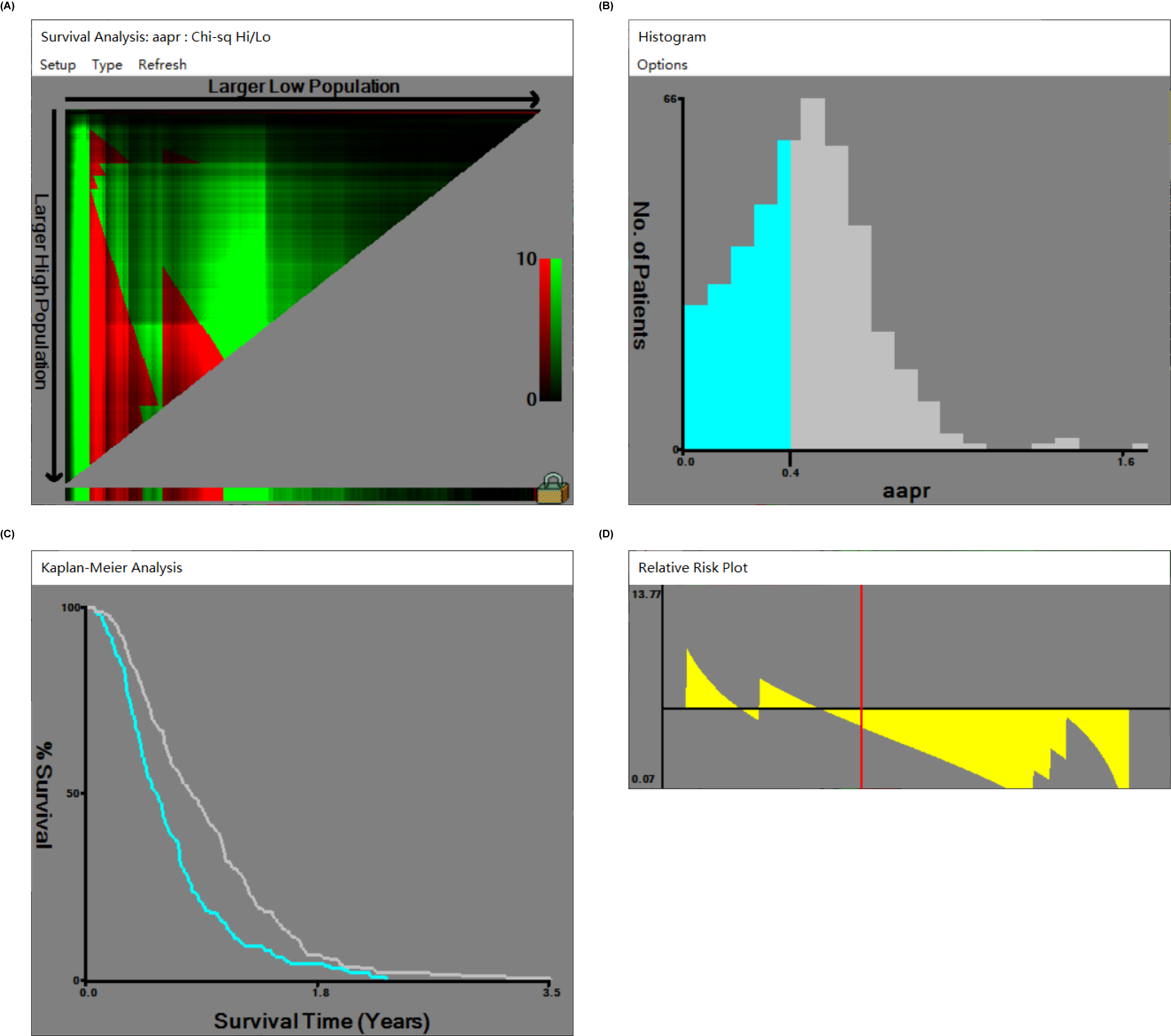

Supplement: Supplementary file 1 — Additional file 1: Figure S1. X-tile software for determining the optimal cutoff of AAPR. Chi-square test (A), Histogram of AAPR (B), Kaplan-Meier analysis (C), Relative risk plot (D). [file 12885_2020_7023_MOESM1_ESM.tif]

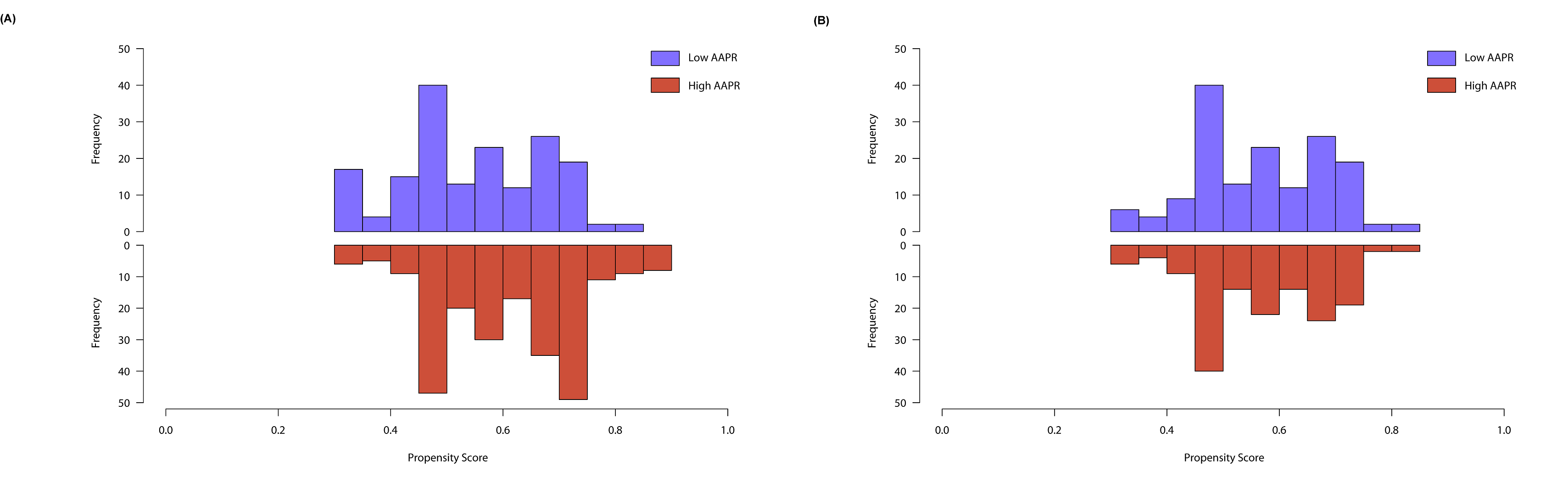

Supplement: Supplementary file 2 — Additional file 2: Figure S2. Mirrored histogram showing distribution and overlapping of propensity score between low and high AAPR groups in unmatched (A) and matched (B) cases. [file 12885_2020_7023_MOESM2_ESM.tif]
